# Correction: Beyond Newton's law of cooling in evaluating magnetic hyperthermia performance: a device-independent procedure

**DOI:** 10.1039/d4na90079k

**Published:** 2024-07-22

**Authors:** Sergiu Ruta, Yilian Fernández-Afonso, Samuel E. Rannala, M. Puerto Morales, Sabino Veintemillas-Verdaguer, Carlton Jones, Lucía Gutiérrez, Roy W. Chantrell, David Serantes

**Affiliations:** a College of Business, Technology and Engineering, Sheffield Hallam University UK sergiu.ruta@shu.ac.uk; b Instituto de Nanociencia y Materiales de Aragón (INMA), CSIC-Universidad de Zaragoza and CIBER-BBN Spain lu@unizar.es; c Department of Physics, University of York UK; d Materials Science Institute of Madrid (ICMM/CSIC) Spain; e nanoTherics Ltd Brookside Farm, Dig Lane Warrington WA2 0SH UK; f Applied Physics Department, Universidade de Santiago de Compostela Spain; g Instituto de Materiais (iMATUS), Universidade de Santiago de Compostela Spain

## Abstract

Correction for ‘Beyond Newton's law of cooling in evaluating magnetic hyperthermia performance: a device-independent procedure’ by Sergiu Ruta *et al.*, *Nanoscale Adv.*, 2024, https://doi.org/10.1039/d4na00383g.

The authors regret that some of the notation used to represent eqn (6)–(8) could be misinterpreted and therefore have been amended.

All calculations were carried out using the 1D heat transport model (eqn (5)). Eqn (6)–(8) indicate the correction to the SLP taking into account the instantaneous losses. The notation is intended to indicate derivatives during heating and cooling rather than absolute values.

In general, the expectation is that the second term in eqn (8) is negative, therefore the correction will be an increase in SLP prediction based on the heating part only. The correct notation is to use square brackets rather than vertical lines around the derivatives, as seen below:6
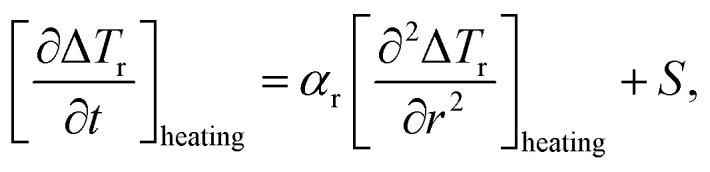
7
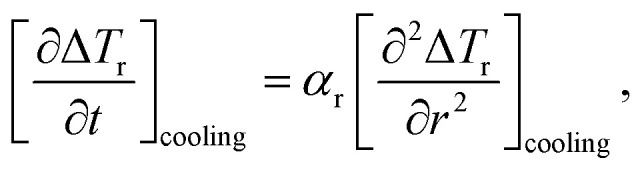
8
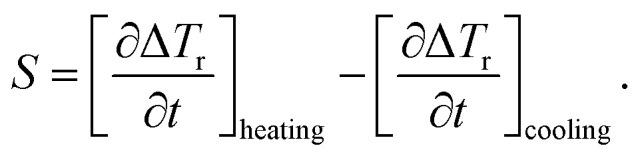


The Royal Society of Chemistry apologises for these errors and any consequent inconvenience to authors and readers.

